# Evaluation of Contrast-Enhanced Intraoperative Ultrasound in the Detection and Management of Liver Lesions in Patients with Hepatocellular Carcinoma

**DOI:** 10.1155/2019/6089340

**Published:** 2019-08-05

**Authors:** Cristina Pace, Vittorio Nardone, Silvia Roma, Fabrizio Chegai, Luca Toti, Tommaso Maria Manzia, Giuseppe Tisone, Antonio Orlacchio

**Affiliations:** ^1^Department of Diagnostic and Interventional Radiology, University Hospital Tor Vergata, Viale Oxford 81, 00133 Rome, Italy; ^2^Department of Surgery, Liver Unit-University Hospital Tor Vergata, Viale Oxford 81, 00133 Rome, Italy

## Abstract

**Aim:**

To evaluate the role of contrast-enhanced intraoperative ultrasound (CE-IOUS) during liver surgery in the detection and management of liver lesions in patients with hepatocellular carcinoma (HCC).

**Materials and Methods:**

From December 2016 to December 2017, 50 patients with HCC, who were candidates for liver resection, were evaluated with intraoperative ultrasound (IOUS). For all patients, MRI and/or CT were performed before surgery. During surgery, IOUS was performed after liver mobilization, and when nodules that had not been detected in the preoperative MRI and/or CT were observed, CE-IOUS scans were carried out with the dual purpose of better characterizing the unknown lesion and discovering new lesions.

**Results:**

In 12 patients, IOUS showed 14 nodules not detected by preoperative MRI and/or CT, before surgery. Out of the 12 lesions, five presented vascular features compatible with those of malignant HCC to the evaluation with CE-IOUS and four of these were simultaneously treated with intraoperative radiofrequency ablation (RFA). The fifth lesion was resected by the surgeon. The remaining nine lesions recognized by IOUS were evaluated as benign at CE-IOUS and considered regenerative nodules. The last diagnosis was confirmed during follow-up obtained by means of CT and/or MRI after 1, 3, 6, or 12 months.

**Conclusion:**

In our experience, CE-IOUS is a useful diagnostic tool in both benign pathologies, such as regenerative nodules, and malignant liver lesions. The advantage of this approach is the possibility of intraoperatively characterizing, based on vascularization patterns, lesions that could not be diagnosed by preoperative imaging, resulting in modification of the surgical therapy decision and expansion of the resection or intraoperative ablation.

## 1. Introduction

Hepatocellular carcinoma (HCC) is the fifth most common malignancy and is one of the main causes of cancer-related death worldwide. This condition is expected to increase further in upcoming years [1, 2]. Modern cross-sectional imaging plays a crucial role in detection and characterization of focal liver lesions, being essential before starting any type of therapy. Moreover, imaging has a significant role during local ablative treatments and on the assessment of the efficacy of percutaneous procedures [3]. The management of HCC involves multiple disciplines including hepatology, surgery, diagnostic and interventional radiology, oncology, and pathology [4, 5].

Nowadays, both contrast-enhanced computed tomography (CE-CT) and magnetic resonance imaging (MRI) with or without liver-specific contrast agent [6] have greatly improved the detection and characterization of liver tumors.

Recent innovations such as contrast-enhanced ultrasound imaging (CEUS) [7, 8] have raised the standards for HCC diagnosis as demonstrated by numerous studies [9, 10].

Hepatic resection is part of the conventional treatment for patients with primary liver cancers; however, the majority of HCCs are not suitable for curative resection at the time of diagnosis [11].

Intraoperative ultrasound (IOUS) is an important tool used during surgical treatment of liver cancers, and particularly in patients with HCC [12, 13], especially when palpable tumors are found intraoperatively and it is mandatory to decide whether resection of malignant lesions is necessary or a lesion appears benign [14].

Proper collaboration between surgeons and interventional radiologists during liver resection in HCC patients is necessary, in order to increase chances of radical treatment in patients with multinodular HCC. Although liver resection represents the first choice of treatment for primary liver cancer, giving the patient the best chance of long-term survival [15], extensive resections of hepatic parenchyma expose patients to the risk of posthepatectomy liver failure (PHLF) associated with a high frequency of postoperative complications, mortality, and an increased length of hospital stay [16].

Thus, radiofrequency ablation (RFA), a widely accepted minimally invasive technique able to destroy tumor effectively and safely [17, 18], should be available during surgical liver resection, in order to save resection of unnecessary healthy hepatic parenchyma.

However, incorrect targeting on imaging could cause inadequate placement of the RFA needle which, in turn, could lead to the need of more treatment sessions or more frequent local recurrence after RFA [19]. It has been demonstrated that CE-IOUS is an accurate diagnostic technique in detecting and characterizing focal liver lesion, and its use in surgical navigation has already been reported in many studies [20–24].

The aim of this study is to evaluate the role of CE-IOUS during liver surgery in the detection and management of liver lesions, as it is known that the technique helps to better characterize the already known lesions. Noteworthy to mention is the fact that not all lesions detected by CE-IOUS in cirrhotic patients with HCC are malignant [21]; therefore, the possibility of intraoperatively characterizing lesions not evident in preoperative imaging is fundamental to guarantee these patients the best therapeutic strategy, performing intraoperative RFA or expanding resection, and more accurate follow-up.

## 2. Materials and Methods

### 2.1. Patients Selection

This is a retrospective study conducted with the approval of the ethics committee, and informed consents were obtained from all patients. From December 2016 to December 2017, 58 patients with chronic liver disease affected by HCC and who were candidates for liver resection were included. Based on the exclusion criteria, 8 patients, lost in follow-up, were excluded from the study. CE-IOUS was performed in 12 cirrhotic patients who presented lesions not evident in preoperative imaging with IOUS evaluation. Cirrhosis was documented by the histological evaluation performed during liver follow-up. The etiology of cirrhosis was hepatitis C in 5 patients, hepatitis B and NASH in 1 patient, alcoholic hepatitis in 2 patients, and cryptogenic hepatitis in 4 patients.

### 2.2. Preoperative Imaging

All patients underwent CT or MRI or both with contrast medium administration before surgery. Contrast-enhanced MRI examinations were performed with a 1.5 T imaging system (Philips Achieva) using T1-weighted (W) turbo spin-echo (TSE), T2-W TSE sequences integrated with fat suppression, dual sequences, and diffusion-weighted imaging (DWI), following an intravenous bolus of 0.1 mmol gadoteridol (ProHance, Bracco SpA, Milan, Italy) per kg of body weight administered at a rate of 2 mL/s and 20 mL of sodium chloride solution. CE-CT scans were performed with a GE Revolution EVO (GE Healthcare, Milan, Italy) pre- and postintravenous injection of 110–130 mL iopamidol (Iopamiro (300 mgI/ml), Bracco SpA, Milan, Italy) with helical scan, 0.6 sec rotation time, pitch 0.9, 120 kV, 250 mA, and image thickness of 2.50 mm. MRI and CT data were acquired in three phases: the hepatic arterial, the portal venous, and the equilibrium. Intraoperative ultrasound patients were treated with open surgery. During surgery, IOUS was performed after liver mobilization and, when nodules that had not been detected in the preoperative MRI and/or CT were observed, CE-IOUS scans were carried out with the dual purpose of better characterizing the unknown lesion and discovering new lesions. CE-IOUS was performed with MyLab Twice (Esaote SpA, Genoa, Italy), equipped with a IOT 342 linear transducer (Top-View) that covering a wider frequency range (3–11 MHz). All lesions were counted and mapped. CE-IOUS was performed with intravenous injection through a peripheral vein of 5 mL ultrasound contrast agent composed of sulphur hexafluoride microbubbles stabilized by a phospholipid shell (SonoVue, Bracco SpA, Milan, Italy). Immediately after the injection, 20 mL saline was injected in the same way. The arterial, portal, and late phases of contrast enhancement were recorded and analyzed. The late phase ended with disappearance of the microbubbles from the circle occurring after 240–360 seconds from the start of the examination [7]. Following the first US contrast medium injection, a full examination of the liver was carried out, segment by segment, to search for new lesions. Only in the case of the patient with two lesions in the two different lobes, two administrations of ultrasound contrast agent, each 2.5 mL, were performed, taking care to wait 6 minutes between one administration and another in order to avoid artifacts. After any RFA, a further CE-IOUS was performed. There were no artifacts or need for flash because a sufficient amount of time elapsed between the CE-IOUS pre- and posttreatment with radiofrequency. The maximum total dose allowed was 3 doses of 5.0 mL.

### 2.3. Histological Analysis and Intraoperative Radiofrequency Thermoablation

For all lesions considered malignant at IOUS and CE-IOUS, biopsy and histological examination were performed. They were treated with surgical resection or with intraoperative RFA. RFA was performed using the RF generator 3000 (Boston Scientific) by positioning the active tip of the needle (LeVeen 14 G) into the lesion with 4 cm of displayed hooks. The RFA procedures were made according to setting of manufacturer up to a final output with roll-off obtained two times. The outcomes of the treatment with RFA were monitored intraoperatively with further ultrasound contrast medium administration and subsequently with follow-up obtained by means of CT and/or MRI after 1, 3, 6, or 12 months.

## 3. Results

Fifty patients were evaluated with IOUS during surgical resection for HCC. In 12 cirrhotic patients, IOUS showed focal lesions not detected by preoperative MRI and/or CT, before surgery. The average age of the patients (six women and six men) was 69.4 years (range 52–78 years).

**Figure 1:**
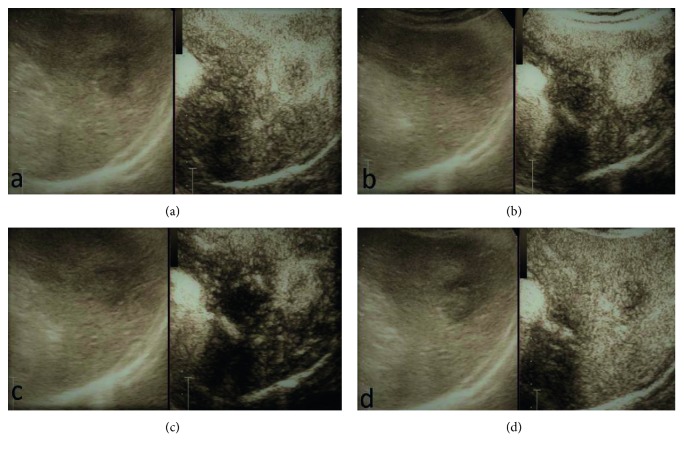
Example of typical vasculature features of CE-IOUS compatible with a malignant nodule of HCC that showed early contrast enhancement and fast washout: (a) early arterial phase; (b) arterial phase; (c) portal phase; (d) late phase.

**Figure 2:**
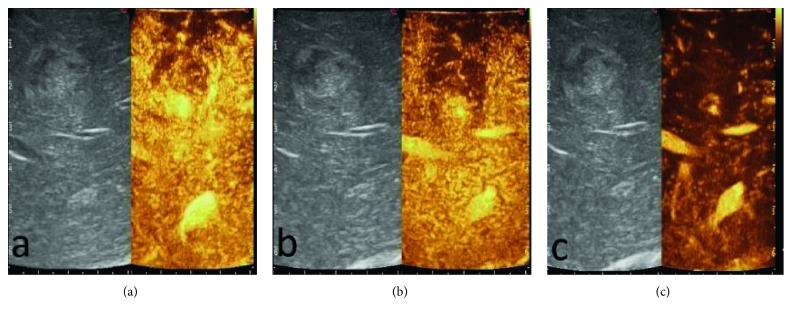
A particular CE-IOUS case showed a peripheral arterial hyperenhancing and hypoechogenic enhancing in the portal and late phases without contrast enhancement in the central area in all phases for hematic and colliquative necrosis component: (a) arterial phase; (b) portal phase; (c) late phase.

**Figure 3:**
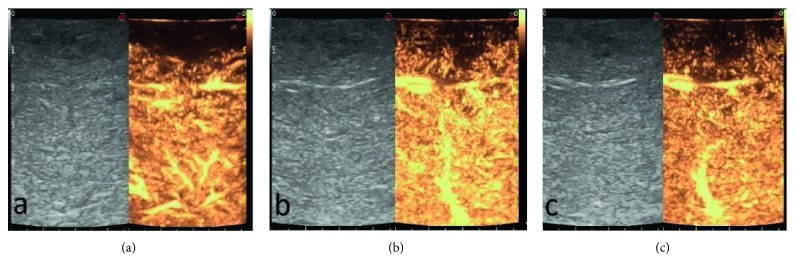
A typical hypoenhancement in all phases of regenerative nodules: (a) arterial phase; (b) portal phase; (c) late phase.

IOUS during hepatic resection of HCC demonstrated 14 nodules. One patient had two lesions in two different lobes, and another patient had two suspected lesions close to each other in the same lobe. These newly detected lesions were evaluated during liver surgery using CE-IOUS, in an attempt to discriminate benign from malignant lesions and decide on treatment in real time. CE-IOUS data were analyzed based on the lesions' wash-in and washout during the arterial, portal venous, and late venous phases. Depending on the vascularization patterns, lesions were characterized as malignant or benign and, if possible, a differential diagnosis was given. Signs of malignancy were considered: arterial phase hyperenhancement followed by late (>60 s) washout [25]. The CE-IOUS allowed us to characterize 14 nodules, with an average size of 10.2 mm (range 7.2 mm–24 mm), five of which present CE-IOUS vascular features compatible with those of malignant HCC (Figures 1 and 2) and were confirmed by biopsy and histological examination. Four of the five malignant lesions detected by CE-IOUS were simultaneously treated with intraoperative RFA, and their complete ablation was intraoperatively evaluated with further ultrasound contrast medium administration. The fifth lesion was resected by the surgeon. The remaining nine lesions recognized by IOUS were evaluated as benign at CE-IOUS and considered regenerative nodules (Figure 3). Since they were small (≤1 cm), it was decided to follow up them. All patients underwent follow-up with CT and/or MRI at 1, 3, 6, and 12 months. The regenerative nodules were confirmed as such, presenting no variation in size and vascularization during follow-up. Also, the complete excision and the absence of disease residues after RFA were confirmed in the follow-up.

## 4. Discussion

Preoperative hepatic imaging diagnosis, such as CT, MRI, and positron emission tomography (PET), has improved considerably in recent years. In 2004, Sahani et al. [26] claimed that MRI is as sensitive as IOUS in depicting hepatic lesions before hepatic resection (86.7% and 94.3%, respectively). Moreover, Huf et al. [27] reported no statistical significance of CEUS and MRI regarding the general differential diagnosis for hepatic tumors. In this study, we highlighted the importance of the presence of interventional radiologist with CE-IOUS and RFA experience during liver resection procedures, in order to perform intraoperative ultrasound, able to ensure optimized liver surgery and able to provide an alternative treatment for unexpected new liver lesions unsuitable for resection. During the long process of the carcinogenesis of HCC, the neovascularization in small lesions may be invisible with the current imaging approaches [28]. Echogenicity of the lesions often changes especially after therapy, embolization, or RFA, which makes it difficult to identify the typical signs of malignancy, e.g., hypoechoic, irregular, and sometimes hypoechoic rim in the periphery of the lesion [29]. Early hypervascularization of HCC lesions in the arterial phase and typical washout of contrast starting in the portal venous phase and continuing in the late phase could be shown only by CEUS. In the CE-IOUS study, HCC lesions are characterized by hyperenhancement during the arterial phase and microbubbles' washout during the portal and late phases [30]. Compared to normal vessels, tumor vessels are tortuous, excessively branched, and short-circuited; thus, overall tumor vasculature appears highly disorganized [31].

The imaging characteristics typical for HCC are difficult to find in small lesions. With the limit of preoperative diagnostic imaging criteria, the Transplantation Network (OPTN)/United Network for Organ Sharing (UNOS) [32] and the Liver Imaging Reporting and Data System (US LI-RADS) [33] suggested not to diagnose, as HCC, lesions <1 cm in diameter.

CE-IOUS can have difficulty in visualizing some regions of the liver even in a mobilized liver (subdiaphragmatic segment VIII). Moreover, special ultrasound devices and a highly experienced examiner are needed to acquire high-quality contrast-enhanced ultrasound scans. In addition, ultrasound is a depth-dependent imaging modality that can reach its limits particularly in overweight patients. CE-IOUS can ensure good visualization of the liver even in overweight patients but is subject to time constraints due to the surgical situation.

This study shows that the use of high definition technique of CE-IOUS with multifrequency probes led to relevant changes in the surgical strategy for malignant liver tumors.

SonoVue® does not impair kidney function as contrast agents used for CE-CT or CE-MRI; therefore, it can be used also in case of reduced creatinine clearance or even kidney failure. The main contraindication for the use of SonoVue® is intolerance for contrast agent component that is very rare, so it is important to specifically exclude this intolerance when obtaining informed consent from the patient.

Results published by Loss et al. [34] showed that in a population of 50 patients, in 28 patients, additional lesions were found using CE-IOUS (mean tumor size of 8 mm, range 4–12 mm). Authors described a change in surgical strategy or the intraoperative application of RFA in 27 patients (54%), resulting in modification of therapy due to additional liver lesions. The largest and most comprehensive analysis of CEUS in the diagnosis of liver tumors is the multicenter prospective DEGUM study [35, 36]. It was able to be shown that CEUS has high diagnostic value for all benign and malignant liver tumor entities. The early detection of small HCC allows new chances for a successful surgery [37].

We observed that CE-IOUS provided an advantage to characterize lesions not detected by presurgical imaging, resulting in changes of the surgical therapy decision and enlargement of the resection or the application of intraoperative ablation. Particularly, we detected 14 new lesions, 5 of them classified as malignant based on CE-IOUS findings and confirmed by biopsy and histological examination. In our experience, the evidence of an additional malignant lesion modified the planned surgical strategy in a patient. In other cases of malignant lesions, in which it was not possible to extend liver resections, radiofrequency ablation (RFA) was done.

RFA has a very important role as an alternative to surgery. Some physicians prefer RFA to surgical resection for the treatment of small HCC even when the patient is eligible for surgery because of the relatively low morbidity and high quality of life [38, 39]. RFA is recommended for the treatment of HCC with a maximum diameter of 3 cm in patients with no more than three tumor masses, in whom surgery is contraindicated [11]. The technical effectiveness of RFA depends on the correct targeting of the nodule on US and adequate placement of the RFA needle [19]. CEUS has been increasingly used for detection, characterization, and planning of therapeutic interventions of liver tumors [9, 10].

As reported, RF ablation guided by a second-generation microbubble-enhanced US may be easier to perform and may be an efficient approach to liver malignancies that are not clearly depicted on B-mode US [11]. CE-IOUS has been reported to be useful also for RFA monitoring [40–42]. In our case, the complete ablation assessed intraoperatively with CE-IOUS was confirmed during follow-up with CT and/or MRI at 1, 3, 6, or 12 months. Also, follow-up confirms that the regenerative nodules maintained benign criteria in imaging over time, and this allows to understand how the IO-CEUS compared to the IO-US allows to effectively distinguish benign from malignant nodules and thus subjecting to the RF treatment a smaller number of lesions than all those evidenced with the IOUS, reducing complications and additional costs in terms of quality of life and economic health.

This study illustrates that intraoperating CE-IOUS allows to accurately detect and characterize liver lesions not evident in preoperative CT and MRI and that it is a useful instrument able to provide additional information during surgery and help in the RFA procedure. The results obtained with CE-IOUS reduce the diagnostic uncertainties and help in guiding the therapeutic choice, increasing the chances of obtaining the nodule's radical resection. Other than the improved characterization of already known and new lesions, CE-IOUS allowed to characterize also benign lesions as nodules of regeneration. This aspect is crucial regarding the clinical-instrumental follow-up of these nodules because, by knowing the regenerative nature of these nodules, it is possible to perform a more targeted follow-up, which is significant in dealing with cirrhotic patients that already need a thorough instrumental monitoring, especially when considering their history of HCC. There were some limitations in this study including its retrospective approach and the single-center design; moreover, the sample was too small to obtain significant statistical data. For these reasons, more studies are needed to further assess this technique.

## 5. Conclusion

This study demonstrated that CE-IOUS is a useful diagnostic tool in both benign and malignant liver lesions. It provides more information than the simple IOUS on the characteristics of the lesion with a dynamic study. In this way, the advantage of this approach is the possibility of intraoperatively characterizing, based on the vascularization patterns, lesions that could not be diagnosed by preoperative imaging, resulting in modification of the surgical therapy decision and expansion of the resection or intraoperative ablation.

## Data Availability

The data used to support the findings of this study are available from the corresponding author upon request.
